# Molecular diversity analysis in hexaploid wheat (*Triticum aestivum* L.) and two *Aegilops* species (*Aegilops crassa* and *Aegilops cylindrica*) using CBDP and SCoT markers

**DOI:** 10.1186/s43141-021-00157-8

**Published:** 2021-04-14

**Authors:** Ghazal Ghobadi, Alireza Etminan, Ali Mehras Mehrabi, Lia Shooshtari

**Affiliations:** Department of plant breeding and Biotechnology, Kermanshah Branch, Islamic Azad University, Kermanshah, Iran

**Keywords:** Genetic diversity, Gene-based markers, Population structure, Polymorphism information content

## Abstract

**Background:**

Evaluation of genetic diversity and relationships among crop wild relatives is an important task in crop improvement. The main objective of the current study was to estimate molecular variability within the set of 91 samples from *Triticum aestivum*, *Aegilops cylindrica*, and *Aegilops crassa* species using 30 CAAT box–derived polymorphism (CBDP) and start codon targeted (SCoT) markers.

**Results:**

Fifteen SCoT and Fifteen CBDP primers produced 262 and 298 fragments which all of them were polymorphic, respectively. The number of polymorphic bands (NPB), polymorphic information content (PIC), resolving power (Rp), and marker index (MI) for SCoT primers ranged from 14 to 23, 0.31 to 0.39, 2.55 to 7.49, and 7.56 to 14.46 with an average of 17.47, 0.34, 10.44, and 5.69, respectively, whereas these values for CBDP primers were 15 to 26, 0.28 to 0.36, 3.82 to 6.94, and 4.74 to 7.96 with a mean of 19.87, 0.31, 5.35, and 6.24, respectively. Based on both marker systems, analysis of molecular variance (AMOVA) indicated that the portion of genetic diversity within species was more than among them. In both analyses, the highest values of the number of observed (Na) and effective alleles (Ne), Nei’s gene diversity (He), and Shannon’s information index (I) were estimated for *Ae. cylindrica* species.

**Conclusion:**

The results of cluster analysis and population structure showed that SCoT and CBDP markers grouped all samples based on their genomic constitutions. In conclusion, the used markers are very effective techniques for the evaluation of the genetic diversity in wild relatives of wheat.

## Background

Based on the International Grains Council’s report (2019) [1], the world needs more one billion tons of wheat for the next 4 years (~ 2024). It seems that this demand is fulfilled through conventional breeding programs [2]. However, there is a main concern among breeders that the genetic background of cultivated genotypes is narrowed by consecutive breeding cycles and remaining variability in its gene pool is inadequate for future breeding programs [3]. Therefore, the expansion of the genetic base of this important cereal is necessary. The genus *Aegilops* as the most important wheat gene pool can contribute to obtaining favorable traits in breeding programs. This genus includes 22 species at the three di-, tetra-, and hexaploid levels as well as with various genetic structures such as the U, M, S, B, D, N, X, and T genomes [4]. Numerous reports have revealed that different *Aegilops* species can be introducing desirable agronomic properties and breeding potential which induce resistance to various biotic and abiotic stresses [5–19]. One of the first fundamental step in each breeding program is the estimate of genetic diversity. Indeed, accurate investigation of the level of genetic diversity can be important in its breeding programs for characterizing ideal parental plants to provide segregating progenies for further basic analysis and selection [20].

Investigation of genetic diversity in wheat and its wild relatives has been done through agro-morphological characters, properties, and molecular markers techniques. Despite the botanical characters and agro-morphological traits being usually used to dissect genetic diversity, they are not completely successful due to environmental influences. In contrast, molecular markers as the genetic tools provide important information regarding the genetic structure and phylogenetic relationships among different plant species. These molecular tools expose genetic differences or similarities in better information without interference from environmental factors [21].

Various molecular marker techniques such as AFLP (amplified fragment length polymorphism), RAPD (randomly amplified polymorphic DNA), SSR (simple sequence repeat), ISSR (inter simple sequence repeats), DArT (diversity arrays technology), etc. are currently available for the evaluation of genetic population analysis, association analysis, and QTL mapping studies. CAAT box–derived polymorphisms (CBDP) and start codon targeted (SCoT) polymorphisms are two new reproducible markers that are based on the short conserved region in plant genes [22, 23]. These techniques have been successfully used in genetic analyses in different plant species [24–32]. The present research is focused on the estimation of genetic diversity and population structure in a set of Iranian bread wheat genotypes and two *Aegilops* species using SCoT and CBDP markers.

## Methods

### Plant materials and DNA extraction

The plant materials consisted of 91 accessions belonging to *Ae. cylindrica*, *Ae. crassa*, and *T. aestivum* L. species. The genetic resources of wild species were collected from the natural habitats in Iran. and the seeds of all accessions were deposited in the Ilam University Gene bank with specific voucher numbers. The genetic composition and gene bank codes are presented in Table 1. Total genomic DNA of investigated accessions was isolated from fresh leaves based on CTAB protocol [33]. The quality of extracted DNA was tested by 0.8% agarose gel electrophoresis.

**Table 1:** List of the 91 investigated *Triticum* and *Aegilops* accessions

No.	Genbank code	Species	No.	Genbank code	Species	No.	Genbank code	Species
1	IUGB-00615	*T. aestivum*	32	NPGBI-365	*Ae. crassa*	62	IUGB-00078	*Ae. cylindrica*
2	IUGB-00597	*T. aestivum*	33	NPGBI-310	*Ae. crassa*	63	IUGB-00090	*Ae. cylindrica*
3	IUGB-00604	*T. aestivum*	34	NPGBI-309	*Ae. crassa*	64	IUGB-00406	*Ae. cylindrica*
4	IUGB-00603	*T. aestivum*	35	NPGBI-2066	*Ae. crassa*	65	IUGB-00258	*Ae. cylindrica*
5	IUGB-00576	*T. aestivum*	36	NPGBI-1589	*Ae. crassa*	66	IUGB-00248	*Ae. cylindrica*
6	IUGB-00618	*T. aestivum*	37	NPGBI-792	*Ae. crassa*	67	IUGB-00388	*Ae. cylindrica*
7	IUGB-01845	*T. aestivum*	38	NPGBI-947	*Ae. crassa*	68	IUGB-01592	*Ae. cylindrica*
8	IUGB-00518	*T. aestivum*	39	NPGBI-1485	*Ae. crassa*	69	IUGB-00202	*Ae. cylindrica*
9	IUGB-00593	*T. aestivum*	40	NPGBI-1508	*Ae. crassa*	70	IUGB-00201	*Ae. cylindrica*
10	IUGB-00570	*T. aestivum*	41	NPGBI-384	*Ae. crassa*	71	IUGB-00406	*Ae. cylindrica*
11	IUGB-00575	*T. aestivum*	42	NPGBI-2112	*Ae. crassa*	72	IUGB-00229	*Ae. cylindrica*
12	IUGB-01846	*T. aestivum*	43	NPGBI-720	*Ae. crassa*	73	IUGB-00090	*Ae. cylindrica*
13	IUGBI-00577	*T. aestivum*	44	NPGBI-2063	*Ae. crassa*	74	IUGB-00270	*Ae. cylindrica*
14	IUGBI-00589	*T. aestivum*	45	NPGBI-911	*Ae. crassa*	75	IUGB-00059	*Ae. cylindrica*
15	IUGB-00573	*T. aestivum*	46	NPGBI-1699	*Ae. crassa*	76	IUGB-00132	*Ae. cylindrica*
16	IUGB-00600	*T. aestivum*	47	NPGBI-587	*Ae. crassa*	77	IUGB-00095	*Ae. cylindrica*
17	IUGB-00578	*T. aestivum*	48	NPGBI-794	*Ae. crassa*	78	IUGB-00062	*Ae. cylindrica*
18	IUGB-00602	*T. aestivum*	49	NPGBI-944	*Ae. crassa*	79	IUGB-01359	*Ae. cylindrica*
19	IUGB-00586	*T. aestivum*	50	NPGBI-2117	*Ae. crassa*	80	IUGB-01238	*Ae. cylindrica*
20	IUGB-00598	*T. aestivum*	51	NPGBI-1742	*Ae. crassa*	81	IUGB-00239	*Ae. cylindrica*
21	IUGB-00515	*T. aestivum*	52	NPGBI-598	*Ae. crassa*	82	IUGB-00078	*Ae. cylindrica*
22	IUGB-01847	*T. aestivum*	53	NPGBI-744	*Ae. crassa*	83	IUGB-00065	*Ae. cylindrica*
23	IUGB-00534	*T. aestivum*	54	NPGBI-1473	*Ae. crassa*	84	IUGB-00391	*Ae. cylindrica*
24	IUGB-00613	*T. aestivum*	55	NPGBI-1522	*Ae. crassa*	85	IUGB-00241	*Ae. cylindrica*
25	IUGB-00590	*T. aestivum*	56	NPGBI-675	*Ae. crassa*	86	IUGB-00153	*Ae. cylindrica*
26	IUGB-00606	*T. aestivum*	57	NPGBI-730	*Ae. crassa*	87	IUGB-00390	*Ae. cylindrica*
27	IUGB-00599	*T. aestivum*	58	NPGBI-689	*Ae. crassa*	88	IUGB-00399	*Ae. cylindrica*
28	IUGB-01840	*T. aestivum*	59	NPGBI-50067	*Ae. crassa*	89	IUGB-00201-S1	*Ae. cylindrica*
29	IUGB-00532	*T. aestivum*	60	NPGBI-50174	*Ae. crassa*	90	IUGB-01592	*Ae. cylindrica*
30	IUGB-00580	*T. aestivum*	61	IUGB-00062	*Ae. cylindrica*	91	IUGB-00388-S1	*Ae. cylindrica*
31	NPGBI-976	*Ae. crassa*						

*IUGB* Ilam University Genebank, *NPGBI* The national Plant Genebank of Iran

### Polymerase chain reaction using SCoT and CBDP markers

A total of 15 SCoT primers(Table 2) were selected based on [21]. Polymerase chain reactions (PCRs) were conducted in a volume of 20 μL and consisted of 2 μL of DNA, 2 μL of each primer, 10 μL master mix PCR (ready-to-use PCR master mix 2×), and 6 μL ddH_2_O. The amplification conditions included an initial denaturation step of 5 min at 94 °C, followed by 45 cycles of 45 s at 94 °C, 1 min at 45 °C, and 3 min at 72 °C with a final extension at 72 °C for 7 min. Produced fragments were separated by gel electrophoresis in 1.5% agarose. In CBDP analysis, 15 primers were designed based on [23], (Table 2). Similar to SCoT assay, each PCR reaction was amplified in a volume of 20 μL and contained 2 μL DNA, 2 μL of each primer, 6 μl double-distilled water, and 10 μl master mix. All reactions were carried out as follows: an initial denaturation step at 94 °C for 5 min, followed by 45 cycles of denaturation at 94 °C for 45 s, primer annealing at 56 °C for 45 s, and primer elongation at 72 °C for 90 s; the final extension at 72 °C was held for 10 min. All products were run on a 1.5% agarose gel. In both systems, all amplified fragments were stained with Safestaine-II and finally photographed using a gel documentation device.

**Table 2:** List of used SCoT and CBDP primers and their calculated informativeness parameters on 91 investigated *Triticum* and *Aegilops*

Primer	Sequence	TB	PB	PIC	Rp	MI
SCoT-2	CAACAATGGCTACCACCC	18	18	0.39	12.04	7.09
SCoT-3	CAACAATGGCTACCACCG	16	16	0.33	8.84	5.29
SCoT-5	CAACAATGGCTACCACGA	16	16	0.26	7.56	4.17
SCoT-6	CAACAATGGCTACCACGC	19	19	0.31	9.69	5.95
SCoT-7	CAACAATGGCTACCACGG	19	19	0.34	9.03	6.42
SCoT-12	ACGACATGGCGACCAACG	14	14	0.37	10.13	5.21
SCoT-13	ACGACATGGCGACCATCG	14	14	0.32	7.93	2.55
SCoT-14	ACGACATGGCGACCACGC	16	16	0.37	10.00	3.70
SCoT-15	ACGACATGGCGACCGCGA	14	14	0.37	14.46	5.22
SCoT-16	CCATGGCTACCACCGGCC	16	16	0.37	9.76	5.90
SCoT-17	CATGGCTACCACCGGCCC	16	16	0.35	11.78	5.63
SCoT-18	ACCATGGCTACCACCGCG	17	17	0.38	10.84	6.43
SCoT-19	GCAACAATGGCTACCACC	21	21	0.36	12.59	7.49
SCoT-20	AACCATGGCTACCACCGC	23	23	0.31	10.79	7.06
SCoT-21	CACCATGGCTACCACCAT	23	23	0.31	11.12	7.19
Mean		17.47	17.47	0.34	10.44	5.69
CBDP-1	TGAGCACGATCCAAT AGC	19	19	0.29	4.69	5.47
CBDP-2	TGAGCACGATCCAATAAT	20	20	0.28	5.65	5.65
CBDP-3	TGAGCACGATCCAAT ACC	21	21	0.31	5.45	6.51
CBDP-4	TGAGCACGATCCAAT AAG	15	15	0.36	6.27	5.33
CBDP-5	TGAGCACGATCCAAT CTA	16	16	0.33	4.12	5.24
CBDP-6	TGAGCACGATCCAAT CAG	15	15	0.36	4.74	5.33
CBDP-7	TGAGCACGATCCAAT CGA	22	22	0.36	6.52	7.96
CBDP-8	TGAGCACGATCCAAT CGG	25	25	0.33	6.95	8.15
CBDP-9	TGAGCACGATCCAAT GAT	23	23	0.29	5.09	6.78
CBDP-10	TGAGCACGATCCAAT GTT	15	15	0.32	4.69	4.75
CBDP-11	TGAGCACGATCCAAT TGC	18	18	0.28	3.82	5.07
CBDP-12	TGAGCACGATCCAATATA	26	26	0.31	7.00	8.07
CBDP-13	TGAGCACGATCCAATGAG	20	20	0.29	5.08	5.84
CBDP-14	TGAGCACGATCCAATGCG	22	22	0.33	5.59	7.36
CBDP-15	TGAGCACGATCCAATTGA	21	21	0.29	4.52	6.05
Mean		19.87	19.87	0.31	5.35	6.24

TB, PB, PIC, Rp, and MI indicate total amplified bands, polymorphic amplified bands, polymorphism information content, resolving power and marker index parameters, respectively

### Data analysis

All the observed bands in SCoT and CBDP profiles were scored as 1 and 0 on the basis of the attendance presence and absence of the amplified fragments, respectively. To determination of efficiency the selected primers, five informativeness indices, such as the number of polymorphic bands (NPB), polymorphism information content (PIC), resolving power (Rp), and marker index (MI), were estimated. Partitioning the genetic diversity among and within species was done through analysis of molecular variance (AMOVA). Genetic variation parameters including the number of observed (*Na*) and effective alleles (*Ne*), Shannon’s information index (*I*), Nei’s gene diversity (*He*), and percentage of polymorphic loci (*PPL*) were calculated for comparing the level of genetic diversity among different species. All genetic parameters were calculated using GenAlEx software [34]. Cluster analysis was computed based on the Jaccard’s dissimilarity matrix to the grouping of the investigated *Aegilops* accessions using DARwin software ver. 6.0.13 [35]. Population structure analysis was carried out using STRUCTURE software [36]. To obtain the optimum number of subpopulations, seven independent runs were determined, so in each run, the values of burn-in period and MCMC factors were 50,000. Then, the results of structure analysis were subjected to an estimate of subpopulations (∆*K*) using the STRUCTURE HARVESTER software [37].

## Results

### SCoT and CBDP polymorphism

All tested SCoT and CBDP primers were polymorphic and reproducible. The summary of estimated informativeness indices for each primer is presented in Table 2. The 15 used SCoT primers amplified 262 distinct fragments, which all of them were polymorphic. The number of bands per primer varied between 14 (SCoT-12, SCoT-13, and SCoT-15) and 23 (SCoT-20 and SCoT-21) with a mean of 17.47 per primer. The average Rp index was 10.44, and primers SCoT-15 and SCoT-5 showed the highest (14.46) and lowest (7.56) values, respectively. The MI index ranged from 2.55 to 7.49 with a mean of 5.69 per primer and the SCoT-19 primer indicated the highest value, while SCoT-13 showed the lowest MI value. PIC index varied between 0.31 and 0.39 with a mean of 0.34. The primer SCoT-1 with the highest value was recognized from others as the informativeness primer, whereas primers SCoT-6, SCoT-20, and SCoT-21 showed the lowest values.

In the CBDP assay, 15 polymorphic primers amplified 298 fragments. Primers CBDP-12 and CBDP-10 amplified the maximum (26) and minimum (15) numbers of polymorphic fragments. Rp index ranged from 3.82 and 6.94 with an average of 5.35 per primer. CBDP-8 and CBDP-11 showed the highest and lowest values for this index than other primers. The MI index varied between 4.74 and 7.96 with an average of 6.24 per primer, and the highest and lowest values were estimated for CBDP-7 and CBDP-10 primers, respectively. The mean of PIC index was 0.31 and it ranged from 0.28 (CBDP-2 and CBDP-11) to 0.36 (CBDP-4, CBDP-6, and CBDP-7) (Table 2).

### Genetic diversity in Aegilops species

**Fig. 1:**
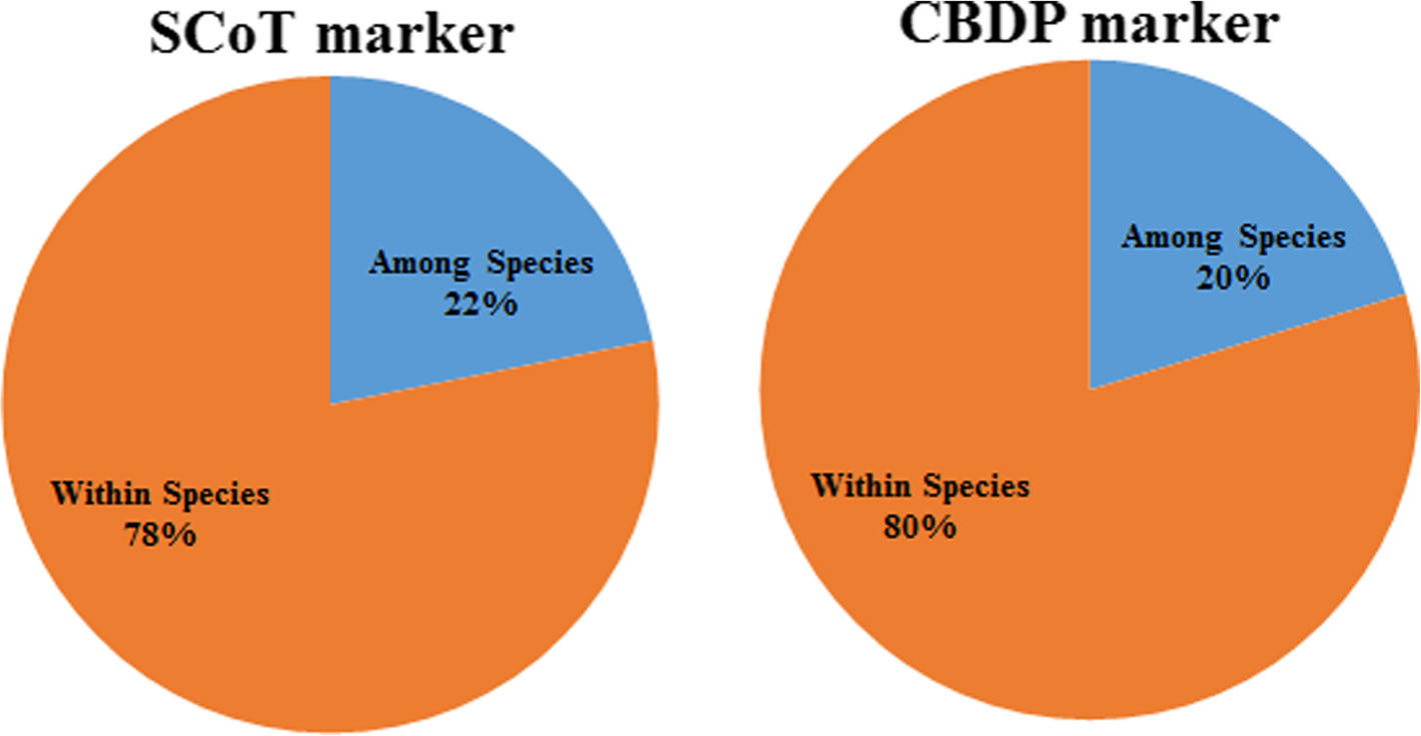
Results of analysis of molecular variance using SCoT and CBDP markers in the 91 investigated wheat’s landraces and wild relatives

The results of the AMOVA analysis are shown in Fig. 1. Based on both marker systems, a significant difference within species was observed. Based on SCoT data, the portion of genetic variance within and between species were 78 and 22%, respectively, while based on CBDP data these portions were 80 and 20%, respectively. Moreover, based on both marker systems, there was a high level of genetic differentiation (*G*_*ST*_) among the studied populations (Table 3). Besides, the values of gene flow (*Nm*) parameter for SCoT and CBDP markers were less than 1, showing a genetic isolation among different species. A summary of the estimated genetic variation parameters based on SCoT and CBDP markers is presented in Table 3. In SCoT analysis, mean values of *Na*, *Ne*, *I*, *He*, and *PPL* were 1.73, 1.35, 0.35, 0.22, and 86.01, respectively. The highest values for *Na* and *PPL* were observed for *T. aestivum* species, while the highest values for *Na*, *Ne*, *I*, and *He* were recorded for *Ae. cylindrica* species. On the other hand, the results based on CBDP data indicated that the average values of all genetic parameters were lower than SCoT data (*Na* = 1.68, *Ne* = 1.33, *I* = 0.33, *He* = 0.21, and *PPL* = 83.78%). The highest values of all parameters were recorded for *Ae. cylindrica* species.

**Table 3:** Estimated genetic parameters in bread wheat and two *Aegilops* using SCoT and CBDP markers

Marker	Species	Sample size	Na	Ne	I	He	PPL	*G*_*ST*_	*Nm*
SCoT	*T. aestivum*	30	1.79	1.35	0.36	0.22	89.31%	0.46	0.78
	*Ae. crassa*	30	1.71	1.33	0.33	0.21	84.35%		
	*Ae. cylindrica*	31	1.71	1.39	0.37	0.24	84.35%		
CBDP	*T. aestivum*	30	1.67	1.36	0.35	0.22	83.56%	0.51	0.82
	*Ae. crassa*	30	1.60	1.29	0.29	0.18	78.86%		
	*Ae. cylindrica*	31	1.79	1.36	0.36	0.23	88.93%		

Na, Ne, I, He, PPL, G_ST_, and Nm indicate the number of observed alleles, number of effective alleles, Shannon’s information index, Nei’s genetic diversity, percentage polymorphism loci, the coefficient of genetic differentiation, and gene flow respectively

### Grouping of *Aegilops* accessions and population structure analysis

**Fig. 2:**
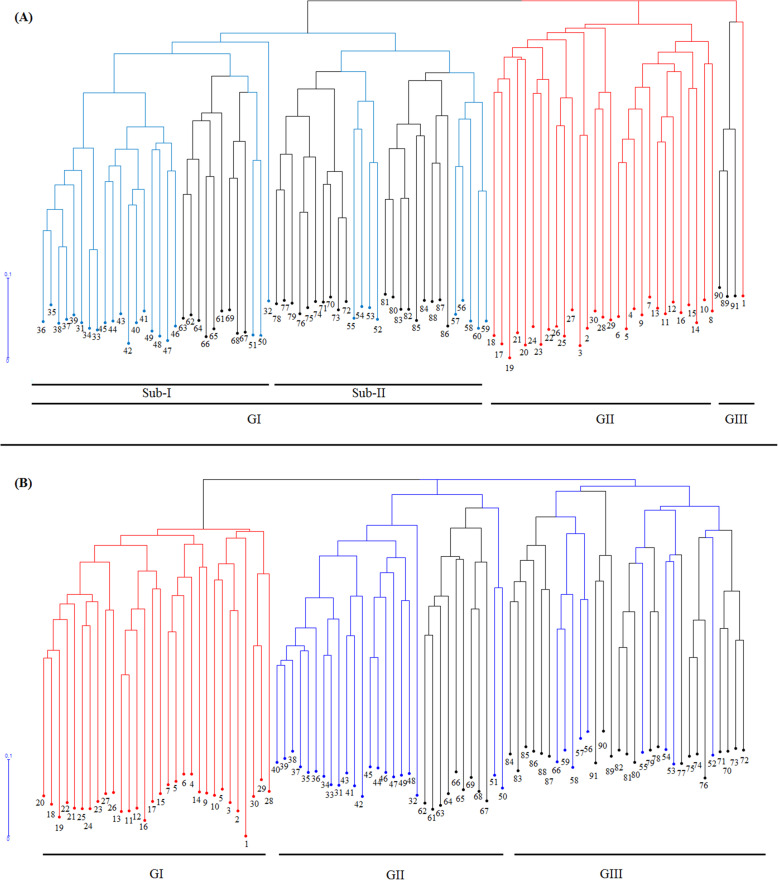
Hierarchical clustering patterns of investigated accessions based on Jaccard’s dissimilarity coefficient using SCoT (panel **a**) and CBDP (panel **b**) markers. Red, black and blue lines represent the *T. aestivum*, *Ae. cylindrica* and *Ae. crassa* accessions, respectively

The dendrogram rendered using the neighbor-joining algorithm (NJ) based on the SCoT data sets clustered all investigated samples into three main groups. The first, second, and third clusters consisted of 58, 29, and 4 samples, respectively (pairwise genetic distance coefficients are not shown). The first cluster (GI) was further divided into two sub-clusters (sub-I and sub-II). Sub-I included 21 accessions from *Ae. crassa* and 9 accessions from *Ae. cylindrica*, while sub-II consisted of 9 and 22 accessions from *Ae. cylindrica* and *Ae. crassa*, respectively. All *T. aestivum* accessions (except no. 1) were placed in the second cluster (GII). Three samples from *Ae. cylindrica* (nos. 89, 90, and 91) along with one sample of *T. aestivum* (no. 1) created the third cluster (GIII) (Fig. 2a). The dendrogram obtained using the CBDP data set indicated that all *Aegilops* samples were grouped into three main clusters. The first cluster (GI) embraced all bread wheat accessions. The second cluster (GII) consisted of 22 samples from *Ae. cylindrica* along with 21 samples from *Ae. crassa* species. The remaining samples from *Ae. cylindrica* and *Ae. crassa* were grouped in the third cluster (GIII) (Fig. 2b).

**Fig. 3:**
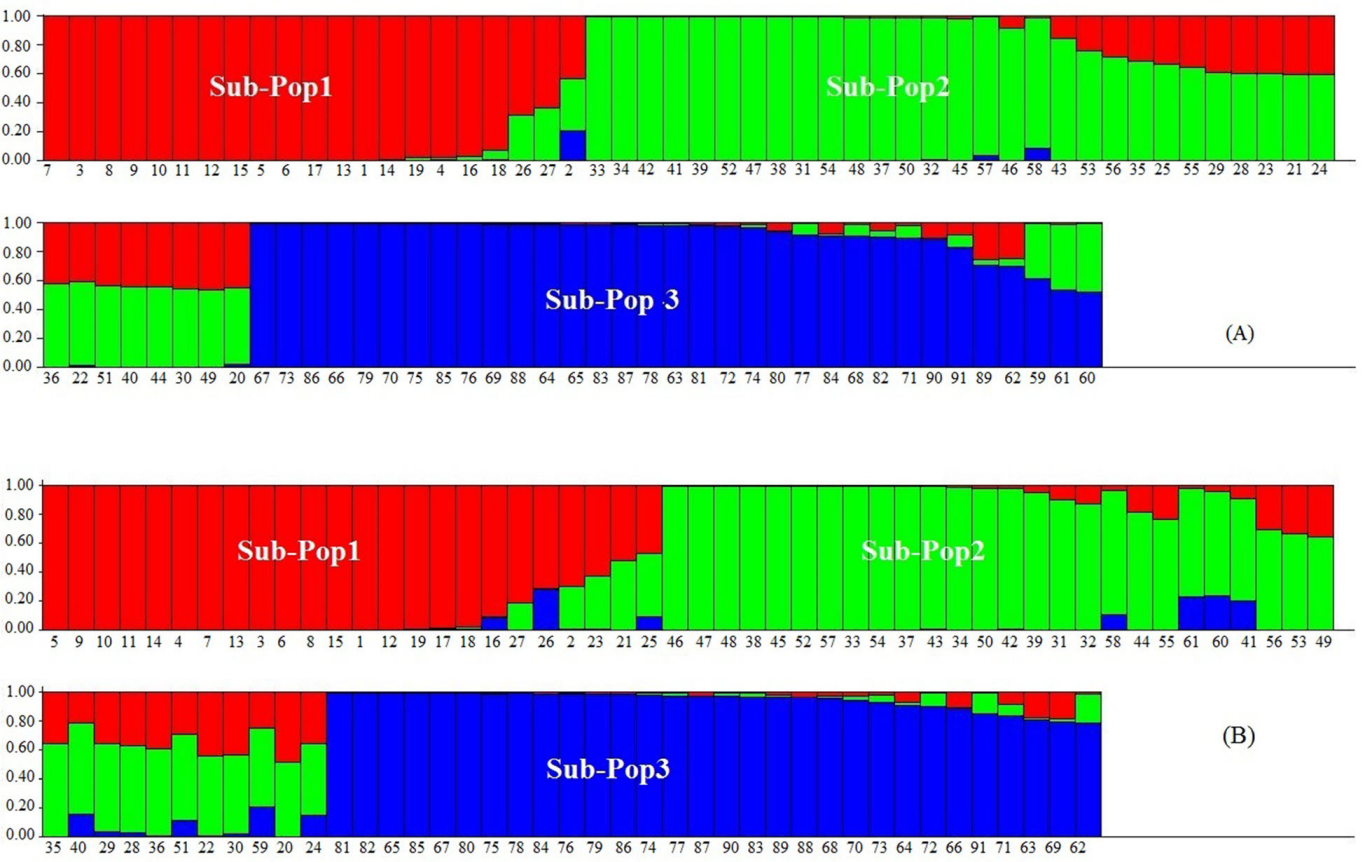
Population structure of 91 *Triticum* and *Aegilops* accessions using SCoT (panel **a**) and CBDP (panel **b**)

**Fig. 4:**
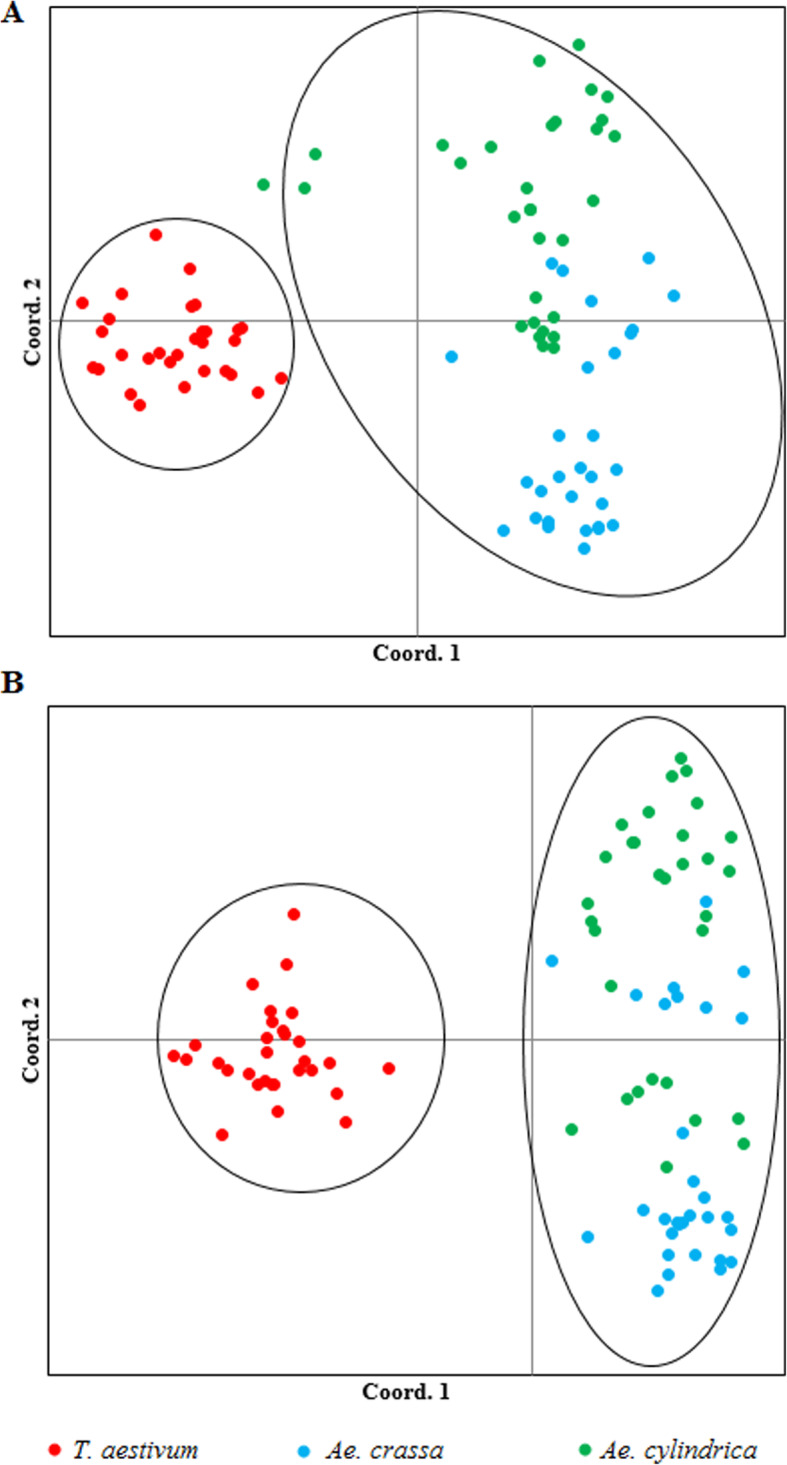
Principal coordinate analysis (PCoA) using SCoT (panel **a**) and CBDP (panel **b**)

In the population structure analysis, the maximum ΔK for both data sets were observed at *K* = 3, with accessions falling into three subpopulations (Fig. 3a). In both analyses, the threshold level of membership for each sample in subpopulations was determined ≥ 0.5. Based on SCoT data, 20 samples of *T. aestivum* created the first subpopulation. All accessions of *Ae. crassa* except nos. 59 and 60 along with nine samples belonging to *T. aestivum* were clustered into the second subpopulation. Two samples from *Ae. crassa* (nos. 59 and 60) and all *Ae. cylindrica* were the third subpopulation. One sample (accession no. 2 from *T. aestivum*) was categorized into an admixed group (Fig. 3a). In CBDP analysis, the optimum number of subpopulations was revealed to be *K* = 3, which indicated that all of the samples can be grouped into three main subpopulations with an admixed group. Out of 30 samples from *T. aestivum* species, 22 samples were placed in subpopulation I, five samples (Nos. 20, 22, 28, 29, and 30) fell into subpopulation II, and three samples (nos. 21, 24, and 25) along with one sample from *Ae. crassa* species (no. 59) were placed in the admixed group, respectively. All samples from *Ae. crassa* were separated from other samples and created subpopulation II. However, one sample from *Ae. cylindrica* was categorized in this subpopulation. Finally, the remaining *Ae. cylindrica* (30 samples) were assigned to subpopulation III (Fig. 3b). The results obtained by cluster analysis and population structure are generally supported by the principal coordinate analysis (PCoA). As shown in Fig. 4, all investigated samples were grouped into two main clusters using SCoT and CBDP markers. In both biplots, all accessions belonging to *T. aestivum* species were placed into the same cluster; however, all *Ae. cylindrica* and *Ae. crassa* fell into the same cluster together.

## Discussion

Increasing the crop adaptability to climate change and ensuring food security for the next century are two critical scenarios which reveal the importance of genetic diversity in crop wild relatives. Among cereal crops, wheat has a rich gene pool, including many wild relatives with various genomic constructions. This feature has enabled wheat breeders to use them as a main source of important agronomic characters and ideal genes which are involved in tolerance to different biotic and abiotic stresses [38]. Therefore, investigation of molecular variability in wild relatives of wheat is a key task in exploring novel genes or even alleles for future breeding programs [39]. Molecular analysis study using DNA-based markers is an efficient approach to estimate genome diversity and population structure that has been used repeatedly in many plants. In the current study, CBDP and SCoT marker techniques served to investigate genetic diversity between and within two *Aegilops* species along with local bread wheat genotypes. All amplified fragments using both marker systems were polymorphic, which confirmed that the used markers are a powerful tool for further genetic diversity analyses and classify the investigated samples (Table 2). Pour-Aboughadareh et al. [40] used SCoT markers to analyze polymorphism of four *Triticum* species including *T. aestivum*, *T. durum*, *T. urartu*, and *T. boeoticum* and obtained 97.59% of polymorphism fragments. Analogous works on wild relatives of bread wheat and different *Aegilops* species were conducted by Pour-Aboughadareh et al. [29] and Etminan et al. [41] and these researchers reported the high level of polymorphism using SCoT and CBDP markers. In addition to percentage polymorphism, resolving power (Rp) and polymorphism information content (PIC) are the important indices of marker usefulness used for comparison efficiency of markers for genetic analyses [29]. PIC depends on the number of detectable alleles and is described as the probability of a primer in identifying polymorphism between samples. On the other hand, Rp shows the discriminatory ability of the used primers to produce informative fragments [42]. Average amounts of PIC and RP confirmed the usefulness of the selected primers for analysis of genetic diversity and grouping the samples belonging to the different species of the *Triticum* and *Aegilops* genera (Table 2). Likewise, Etminan et al. [43] investigated accessions of the *T. durum* and obtained a PIC of 0.31 and Rp of 9.16 using CBDP markers. Also, in another study, these authors used fifteen CBDP primers for dissection of molecular variability in different *Aegilops* and *Triticum* species and reported a high level of polymorphism and discriminatory of the used markers (PIC = 0.47 and Rp = 11.19). Nowak et al. [2] analyzed three *Aegilops* species (*Ae. crassa*, *Ae. neglecta*, and *Ae. juvenalis*) using REMP and ISSR markers. The authors indicated that the used markers were efficient systems for evaluating the genetic diversity and also reported that the *Aegilops* species have a high level of genome variability which can serve as an ideal gene pool for discovering useful genes.

The result of AMOVA revealed that genetic diversity observed within species (SCoT = 78% and CBDP = 80%) is more to that found among them (SCoT = 22% and CBDP = 80%), suggesting all accessions in each species have a wide genetic differentiation (Fig. 1). This finding is in accordance with those of the previous reports that showed the high level of diversity in *Aegilops* species through different DNA markers [2, 29, 41, 44–48]. Our results indicate the accessions from three different species are genetically different from each other. As shown in Table 3, the highest values of the genetic variation indices (especially Na, Ne, I, and He) were observed for *Ae. cylindrica* species using both marker systems. The higher level of diversity in this species might be referred to as the frequency of allelic variation of this species being affected by different climatic conditions [2]. Several studies considered *Ae. cylindrica* as novel sources of tolerance to abiotic stresses for further wheat breeding programs [49]. Pour-Aboughadareh et al. [29] reported that *Ae. cylindrica* has the highest level of genetic diversity among the evaluated *Aegilops* species, whereas the lower level of diversity belonged to *Ae*. *crassa*, which was in accordance with our findings in this study. However, this result disagrees with Etminan et al. [41], so these authors reported a high level of genetic diversity in *T. aestivum* and *Ae. crassa* then *Ae. cylindrica* using CBDP markers. These contradictions could be referred to the primer’s sequences, the geographical origins, or sample size of the tested accessions. Khodaee et al. [50] also reported a high level of genetic diversity among the Iranian *Ae. triuncialis* accessions using ISSR, CBDP, and SCoT molecular markers and confirmed that all the three marker systems can provide a comprehensive pattern of the genetic diversity in *Ae. triuncialis* germplasm.

In SCoT and CBDP analyses, clustering patterns were consistent with the results of population structure analysis. In both analyses, all investigated accessions were clustered based on their genomic structure with a minor admixture (Figs. 2, 3, and 4). Previously, a similar grouping pattern was observed for accessions from different *Aegilops* and *Triticum* species by Pour-Aboughadareh et al. [29] and Etminan et al. [41]. These authors reported SCoT and CBDP markers group species based on their genetic backgrounds and the obtained groups or subpopulations approximately confirm their taxonomic classification.

## Conclusion

Preservation of the highest possible level of genetic diversity is one of the main goals of genetic resource conservation programs and assessment of genetic diversity using reliable methods provides useful information for the management of genetic resources and crop improvement programs. Our results revealed high polymorphism in the investigated Iranian wheat germplasm from different *Triticum* and *Aegilops* species. The molecular analysis of genetic diversity in the tested species showed a high level of genome variability in *Ae. cylindrica* species. Based on the results of AMOVA, genetic diversity observed within species was more than that found among them suggesting all accessions in each species have a wide genetic differentiation. In addition, based on obtained results, SCoT and CBDP markers were very effective techniques for the evaluation of the genetic diversity and phylogenetic studies in wheat germplasm. These results revealed that these two different gene-targeted molecular markers can be used as reliable techniques for detecting the levels of DNA polymorphism and genetic relationship.

## Data Availability

All data generated or analyzed during this study are included in this published article.

## Abbreviations

*T*: *Triticum*
*Ae*: *Aegilops*
*CBDP*: CAAT box–derived polymorphism
*NBP*: Number of polymorphic bands
*I*: Shannon index
*ISSR*: Inter-simple sequence repeats
*MI*: Marker index
*PCoA*: Principal coordinate analysis
*PIC*: Polymorphism information content
*Rp*: Resolving power
*SCoT*: Start codon targeted
*UPGMA*: Unweighted pair group method with arithmetic mean algorithm
*AMOVA*: Analysis of molecular variance
Na: Number of observed alleles
*Ne*: Number of effective alleles
*He*: Nei’s gene diversity
*AFLP*: Amplified fragment length polymorphism
*RAPD*: Randomly amplified polymorphic DNA
*SSR*: Simple Sequence Repeat
*DArT*: Diversity arrays technology
*QTL*: Quantitative trait loci
